# Occurrence and Severity of Mental Health Disorders Among Patients Treated for Malignant Neoplasms: A Narrative Review with Preliminary Quantitative Synthesis

**DOI:** 10.3390/jcm15197589

**Published:** 2026-09-30

**Authors:** Robert Jan Łuczyk, Dorota Weber, Marta Łuczyk, Kamil Sikora, Anna Charuta

**Affiliations:** 1 Institute of Health Sciences, Faculty of Medical and Health Sciences, University of Siedlce, 08-110 Siedlce, Poland; anna.charuta@uws.edu.pl; 2 Faculty of Health Sciences, Lublin University of Technology, WSEI in Lublin, Projektowa 4, 20-209 Lublin, Poland; dorota.weber@wsei.pl; 3 Long-Term Care Nursing Department, Chair of Preventive Nursing, Faculty of Health Sciences, Medical University of Lublin, Ul. Chodźki 7, 20-093 Lublin, Poland; marta.luczyk@umlub.edu.pl; 4 Department of Internal Medicine and Internal Nursing, Chair of Preventive Nursing, Faculty of Health Sciences, Medical University of Lublin, Ul. Chodźki 7, 20-093 Lublin, Poland; kamil.sikora@umlub.edu.pl

**Keywords:** cancer, oncology, depression, anxiety, post-traumatic stress disorder, insomnia, suicidal ideation, psycho-oncology, meta-analysis, narrative review

## Abstract

**Background/Objectives:** A cancer diagnosis and its subsequent treatment constitute one of the most psychologically demanding experiences a patient can face, and decades of psycho-oncology research have documented an elevated burden of depression, anxiety, and related conditions in this population. Reported prevalence figures nonetheless vary enormously between studies—a 2011 meta-analysis by Mitchell and colleagues, for instance, arrived at pooled depression estimates roughly half those reported in some subsequent work—complicating clinical decisions about who should be screened and how intensively. The present narrative review with preliminary quantitative synthesis set out to chart this variability directly: to assemble a cross-section of the international literature on depression, anxiety, post-traumatic stress disorder (PTSD), insomnia, and suicidal ideation in oncology patients to quantify how far pooled estimates diverge from one another when treatment setting, tumour type, and assessment instrument are allowed to vary freely, and to lay the empirical groundwork for a subsequent, fully systematic review by the author group. **Methods:** Consistent with the exploratory aims stated above, eligible primary studies were located through web-based literature searching and citation chaining rather than exhaustive querying of bibliographic databases; the search was therefore not conducted, and is not reported, as a PRISMA 2020-compliant systematic review. Cross-sectional or baseline cohort studies reporting original prevalence figures for at least one of the five outcomes above, in patients with a histologically confirmed malignancy, including adult and paediatric/adolescent patients, who were undergoing or had completed treatment, using a named validated instrument, were retained. Where two or more studies addressed the same outcome, a random effects pooled proportion (DerSimonian–Laird estimator, Freeman–Tukey double arcsine variance stabilisation) was computed in Python as an illustrative, not definitive, summary statistic, alongside Cochran’s Q and I^2^. **Results:** Twelve studies conducted across nine countries were retained, contributing fourteen outcome-level data points. The illustrative pooled prevalence of depression was 45.6% (95% CI: 29.5–62.3%; k = 3; I^2^ = 91.2%) and of anxiety 44.1% (95% CI: 32.7–55.8%; k = 4; I^2^ = 90.5%)—both considerably above the double-digit figures reported in earlier interview-based meta-analyses, reflecting the self-report screening instruments and, in several instances, lower-income treatment settings represented in the present sample. Pooled PTSD prevalence was 17.0% (95% CI: 7.3–29.7%; k = 3; I^2^ = 91.8%) and pooled insomnia prevalence 38.0% (95% CI: 24.5–52.6%; k = 3; I^2^ = 86.7%). A single Chinese multicentre study of 509 women with advanced breast cancer reported suicidal ideation in 22.8% of respondents; this figure is reported descriptively only, is not pooled, and should not be extrapolated to oncology patients generally. **Conclusions:** Even acknowledging its exploratory character, this synthesis reinforces a conclusion already well established in psycho-oncology: a substantial minority, and on some metrics close to half, of patients receiving cancer treatment report clinically significant psychological symptoms, with figures highest for depression and anxiety in the self-report studies assembled here. The magnitude of between-study heterogeneity observed for every outcome, however, argues strongly against treating any single percentage—ours included—as a stable population estimate; tumour type, treatment phase, country income level, and instrument choice all plausibly move the figure by a considerable margin, and disentangling these influences is the explicit purpose of the fully systematic review the author group is now undertaking; the present figures should be read as hypothesis-generating inputs to that forthcoming systematic review, not as stand-alone prevalence estimates suitable for direct clinical or policy use.

## 1. Introduction

A diagnosis of cancer rarely arrives as purely a physical event. With cancer now diagnosed in an estimated 19 million people worldwide each year [1], it is, for most patients, also the beginning of an extended encounter with uncertainty—about prognosis, about the tolerability of forthcoming treatment, about the practical and financial disruption to daily life—that unfolds over months or years rather than resolving after a single acute episode [2,3]. It is against this backdrop that psycho-oncology has, over roughly four decades, built a substantial evidence base documenting elevated rates of depression, anxiety, adjustment disorder, and related conditions in oncology populations relative to the general population [4], with post-traumatic stress reactions specifically conceptualised as a distinct, if less extensively studied, response to the perceived life threat of a cancer diagnosis [5].

Quantifying exactly how elevated has proven surprisingly difficult. The landmark meta-analysis by Mitchell and colleagues, pooling 94 interview-based studies across oncological, haematological, and palliative care settings, arrived at a combined prevalence of 16.5% for syndromal depression by DSM or ICD criteria and 9.8% for anxiety disorders [6]—figures that many subsequent reviews have cited as a benchmark, yet that a later meta-analysis restricted to lower- and lower-middle-income countries found to be roughly 30% too low, reporting 21% for major depression and 18% for anxiety disorders in that setting [7]. A separate synthesis focused specifically on depression reported pooled prevalence ranging from 8% to 24% depending solely on which screening or diagnostic instrument the underlying studies had used [8], while a meta-analysis restricted to ovarian cancer found depression prevalence shifting from roughly 25% before treatment to under 13% afterward [9]. Sleep disturbance follows a similar pattern: a 2022 synthesis of 160 studies (N = 46,279) reported an overall prevalence of 60.7%, with near-maximal between-study heterogeneity (I^2^ = 96.4%) [10]. Post-traumatic stress symptoms specifically attributable to the cancer experience have received comparatively less quantitative synthesis, though available estimates range from roughly 5% to over 15% depending on whether a checklist cut-off or a structured clinical interview is applied [11].

Two observations follow from this body of work. First, the true burden of psychological morbidity in oncology populations is not seriously in doubt—every major synthesis to date situates it well above general population base rates. Second, and more consequentially for both clinical screening policy and future research design, the precise magnitude of that burden appears to depend heavily on factors that vary from study to study: which instrument was used, at what point in the treatment trajectory patients were assessed, which tumour type was studied, and in which country and income setting the study was conducted. Existing reviews have generally examined these sources of variation one at a time—by instrument [8], by income setting [7], by tumour site [9]—rather than assembling a single dataset spanning multiple outcomes and multiple candidate moderators simultaneously.

The present work takes a preliminary step in that direction. Rather than attempting an exhaustive systematic search at this stage, we assembled a purposive, internationally diverse sample of primary studies spanning five distinct mental health outcomes—depression, anxiety, PTSD, insomnia, and suicidal ideation—in patients undergoing or having completed cancer treatment, computed illustrative pooled estimates for each, and examined the pattern of heterogeneity that resulted. Our aims were threefold: (1) to demonstrate, using real extracted data, the scale of divergence that arises even within a modestly sized, non-exhaustive sample of studies; (2) to identify candidate explanations for that divergence that a subsequent, fully systematic review should test formally; and (3) to situate this exploratory work within the authors’ broader programme of psycho-oncology research in Poland, where population-specific prevalence data remain comparatively sparse in the international literature.

## 2. Materials and Methods

### 2.1. Positioning of This Review

This article is a narrative review with an embedded, illustrative quantitative synthesis; it does not claim, and should not be read as satisfying, the PRISMA 2020 standard for systematic reviews [12]. The rationale for this scope is practical rather than principled: assembling the exhaustive, reproducible, multi-database evidence base that psycho-oncology’s decades of accumulated literature would require is a substantial undertaking, and the authors judged it more useful to first characterise, with genuine (if incomplete) data, the shape and likely magnitude of cross-study variability, before committing to the full systematic search. A companion protocol specifying the complete search strategy for that subsequent systematic review has been drafted by the author group and is available on request.

### 2.2. Eligibility Criteria

Primary studies were eligible if they reported an original prevalence figure—derived from a named, validated screening or diagnostic instrument—for depression, anxiety, PTSD, insomnia, or suicidal ideation in a sample of patients with a histologically confirmed malignant neoplasm, including adult and paediatric/adolescent patients, assessed at any point from diagnosis through post-treatment survivorship. Both self-report screening instruments (e.g., HADS, PHQ, GAD-7, ISI) and structured clinical interviews or checklist-based diagnostic tools (e.g., PCL, IES) were accepted, on the understanding that instrument type would itself be examined as a candidate source of heterogeneity rather than treated as an exclusion criterion. Studies restricted to caregivers, healthy controls, or non-cancer comparison groups were excluded, as were case reports, editorials, and reviews not reporting original data. No restriction was placed on cancer type, country, or publication date; English- and Polish-language publications were both eligible.

### 2.3. Literature Identification

Candidate studies were located through iterative keyword searching of general academic search engines, cross-referencing of large secondary meta-analyses identified early in the process (which in several cases pointed toward their own included primary studies), and follow-up searches once a relevant research group or patient cohort was identified in an initial source. This process was carried out by a single reviewer with AI-assisted search support, did not involve direct querying of PubMed/MEDLINE, Scopus, Embase, PsycINFO, Web of Science, or CINAHL through their native interfaces, and did not apply a fixed Boolean search string of the kind that will be specified in the companion systematic review protocol. The resulting sample should accordingly be treated as a convenience sample illustrating the range of the literature rather than as a complete inventory of it; no PRISMA flow diagram is presented, since the identification process described here does not generate the database-level counts that such a diagram is intended to summarise.

### 2.4. Data Extraction

For each eligible outcome within each study, the following were extracted into a structured spreadsheet: country and treatment setting, cancer type, sample size, instrument and cut-off applied, number of cases meeting the threshold, and, where reported, the treatment phase (pre-treatment, on-treatment, post-treatment/survivorship). Extraction was performed by a single reviewer from published abstracts and, where accessible, full texts; entries drawing on incompletely verified full-text detail are flagged accordingly in Table 1.

### 2.5. Quality Considerations

Given the single-reviewer, non-exhaustive nature of the identification process, no formal dual-rater risk-of-bias instrument (e.g., the Joanna Briggs Institute checklist for prevalence studies [13]) was applied across the included set. This represents a deliberate scope limitation rather than an oversight, and it will be remedied in the companion systematic review; readers should treat the pooled figures below as unadjusted for study-level methodological quality.

### 2.6. Statistical Approach

For every outcome with two or more eligible studies, case proportions were stabilised using the Freeman–Tukey double arcsine transformation [14], following the general approach recommended for prevalence meta-analysis [15], and combined under a random-effects model using the DerSimonian–Laird between-study variance estimator [16]. Between-study heterogeneity was summarised with Cochran’s Q and the I^2^ statistic, interpreted against the conventional benchmarks of roughly 25%, 50%, and 75% for low, moderate, and high inconsistency [17,18]. All computation was performed in Python (NumPy) ver. 2.5.3; the definitive analysis for the planned systematic review will instead use the metafor package in R [19] or the metaprop command in Stata ver. 18 [20], both of which implement the variance-weighted back-transformation recommended for prevalence meta-analysis [21] rather than the simplified sin^2^ approximation used here. Because no outcome in the present sample reached ten contributing studies, formal small-study-effect testing (Egger’s test [22]) and meta-regression were not attempted; plausible moderators—cancer type, treatment phase, country income level, and instrument—are instead discussed qualitatively in Section 4.

## 3. Results

### 3.1. Composition of the Retrieved Sample

Twelve unique primary studies met the eligibility criteria described in Section 2.2, together contributing fourteen outcome-level observations across five mental health domains: three each for depression and PTSD, four for anxiety, three for insomnia, and one for suicidal ideation (Table 1). Several studies contributed data on more than one outcome from the same patient sample. The included studies were conducted in India, Bangladesh, Portugal, China, Italy, Oman, the United States, South Korea, and Tunisia, and covered breast, haematological, testicular, paediatric, and mixed/unspecified cancer cohorts, at treatment phases ranging from active chemotherapy to multi-year survivorship. As set out in Section 2.3, this yield reflects a targeted, non-exhaustive identification strategy; the planned systematic review is expected to identify a substantially larger evidence base, particularly from the many single-country prevalence studies not captured by the present search approach.

**Table 1:** Studies contributing to the preliminary synthesis (k = 14 outcome-level observations from 12 unique studies). Where a study reported a threshold other than “caseness” on the full clinical scale (e.g., a single self-report item for suicidal ideation), this is noted in the Outcome column.

Study	Country	Cancer Type	N	Instrument	Outcome	Cases (n)	Prevalence (%)
Dadheech et al. [23]	India	Breast	140	PHQ-2	Depression	53	37.9
Dadheech et al. [23]	India	Breast	140	GAD-2	Anxiety	47	33.6
Islam et al. [24]	Bangladesh	Breast (metastatic)	95	HADS	Depression (mod.-severe)	35	36.9
Islam et al. [24]	Bangladesh	Breast (metastatic)	95	HADS	Anxiety (mod.-severe)	42	44.2
Radhakrishnan et al. [25]	India	Mixed	162	HADS	Depression (borderline+)	100	61.7
Radhakrishnan et al. [25]	India	Mixed	162	HADS	Anxiety (borderline+)	99	61.1
Lopes et al. [26]	Portugal	Breast	506	HADS	Anxiety (baseline)	192	38.0
Liu et al. [27]	China	Haematological	225	PCL-C	PTSD	24	10.7
De Padova et al. [28]	Italy	Testicular	209	IES	PTSD (probable)	23	11.0
Al-Saadi et al. [29]	Oman	Mixed (paediatric)	101	PTSD subscale	PTSD	33	32.6
Hoang et al. [30]	USA	Mixed (chemotherapy)	213	ISI	Insomnia	91	42.8
Ruiz-Marin et al. [31]	South Korea	Mixed	130	ISI	Insomnia (mod.-severe)	32	24.7
Abdelkefi et al. [32]	Tunisia	Lung	49	ISI	Insomnia (mild-mod.)	24	49.0
Xu et al. [33]	China	Breast (advanced)	509	self-report item	Suicidal ideation	116	22.8

### 3.2. Study Characteristics

Table 1 lists the twelve included studies with their country, cancer type, sample size, instrument, and outcome-specific prevalence.

### 3.3. Depression and Anxiety

**Figure 1:**
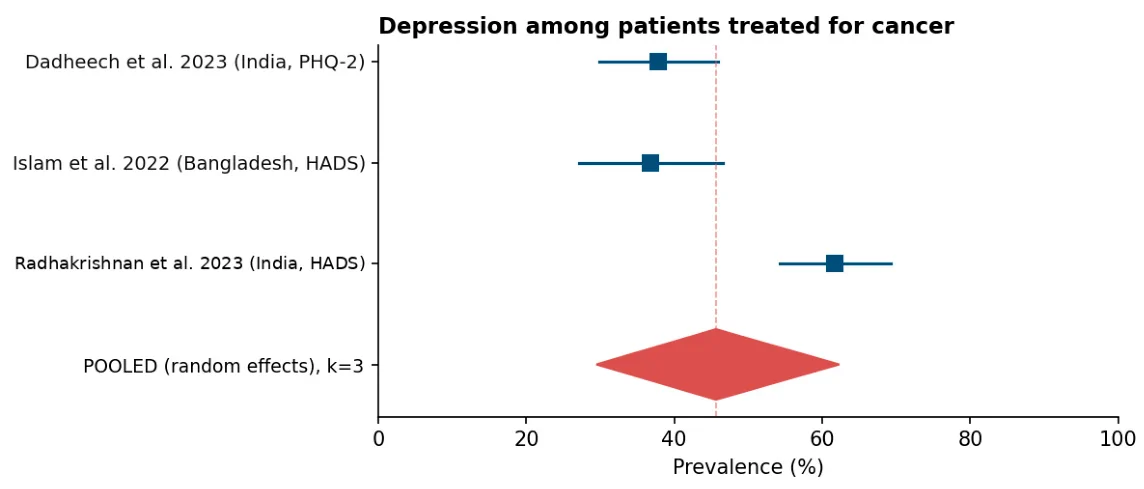
Illustrative forest plot of depression prevalence in oncology patients (random effects model; preliminary sample, k = 3) [23,24,25].

Three studies (total N = 397) reported depression prevalence, using two different screening approaches (a two-item PHQ-2 screen in one Indian sample [23]; the depression subscale of the Hospital Anxiety and Depression Scale, HADS, in the Bangladeshi and second Indian sample [24,25]). The illustrative pooled prevalence was 45.6% (95% CI: 29.5–62.3%; Q = 22.84, df = 2, *p* < 0.001; I^2^ = 91.2%; τ^2^ = 0.020) (Figure 1), with point estimates ranging from 36.9% in the Bangladeshi metastatic-breast-cancer sample [24] to 61.7% in the mixed cancer Indian sample using the full HADS “borderline-or-above” threshold [25]—a threshold appreciably more permissive than the diagnostic criteria applied in classic interview-based meta-analyses [6].

**Figure 2:**
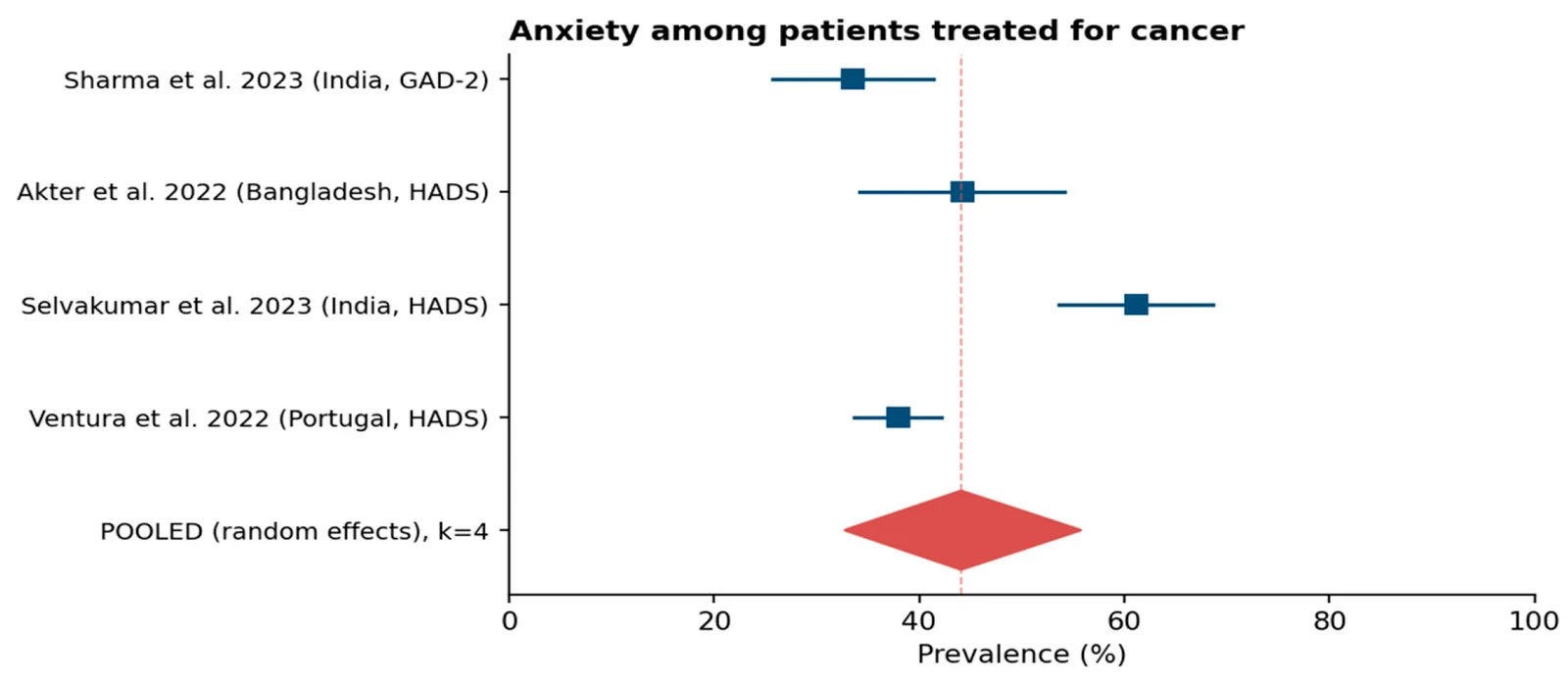
Illustrative forest plot of anxiety prevalence in oncology patients (random effects model; preliminary sample, k = 4) [23,24,25,26].

Four studies (total N = 903) reported anxiety prevalence, adding a large Portuguese breast cancer cohort assessed at baseline (pre-treatment) to the three studies contributing depression data [23,24,25,26]. The pooled prevalence was 44.1% (95% CI: 32.7–55.8%; Q = 31.66, df = 3, *p* < 0.001; I^2^ = 90.5%; τ^2^ = 0.013) (Figure 2). The Portuguese estimate (38.0%) [26] sat close to the pooled figure despite deriving from the largest and, on its face, most representative sample in the set, whereas the Indian mixed cancer estimate (61.1%) [25] again stood out as the high outlier, mirroring the pattern seen for depression in the same study and suggesting that at least part of the elevation in that particular sample reflects study-level rather than purely national or instrument-level factors.

### 3.4. Post-Traumatic Stress Disorder

**Figure 3:**
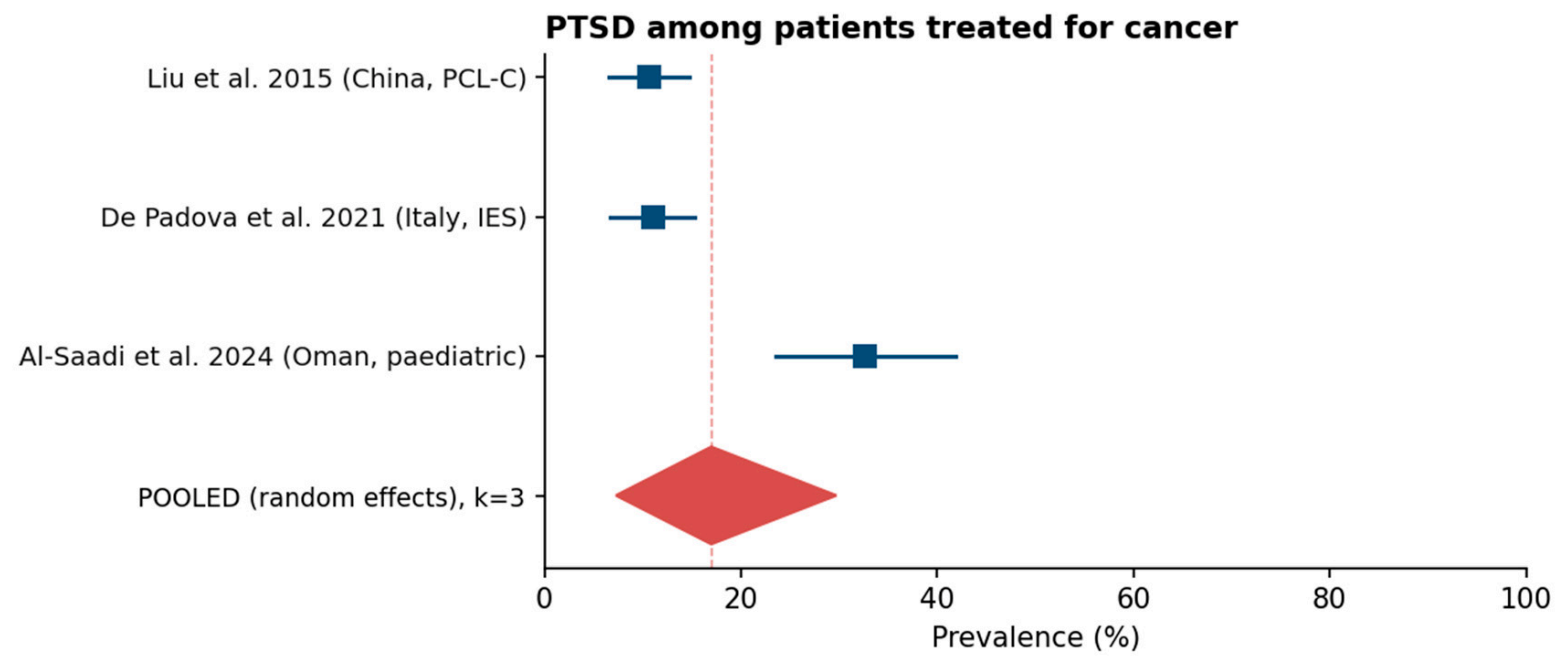
Illustrative forest plot of PTSD prevalence in oncology patients (random effects model; preliminary sample, k = 3) [28,29,34].

Three studies (total N = 535) reported PTSD prevalence, spanning a haematological malignancy cohort in China [27], a long-term testicular cancer survivor cohort in Italy [28], and a paediatric/adolescent oncology cohort in Oman [29]. The pooled prevalence was 17.0% (95% CI: 7.3–29.7%; Q = 24.38, df = 2, *p* < 0.001; I^2^ = 91.8%; τ^2^ = 0.016) (Figure 3), with the Chinese and Italian adult survivor estimates (10.7% and 11.0%, respectively) [27,28] clustering closely together despite differing tumour types and very different times since diagnosis, while the Omani paediatric estimate (32.6%) [29], assessed within three months of diagnosis, was roughly three times higher—consistent with the broader observation that PTSD symptoms in cancer populations tend to be most prevalent close to diagnosis and to decline over the subsequent treatment course [11,29].

### 3.5. Insomnia

**Figure 4:**
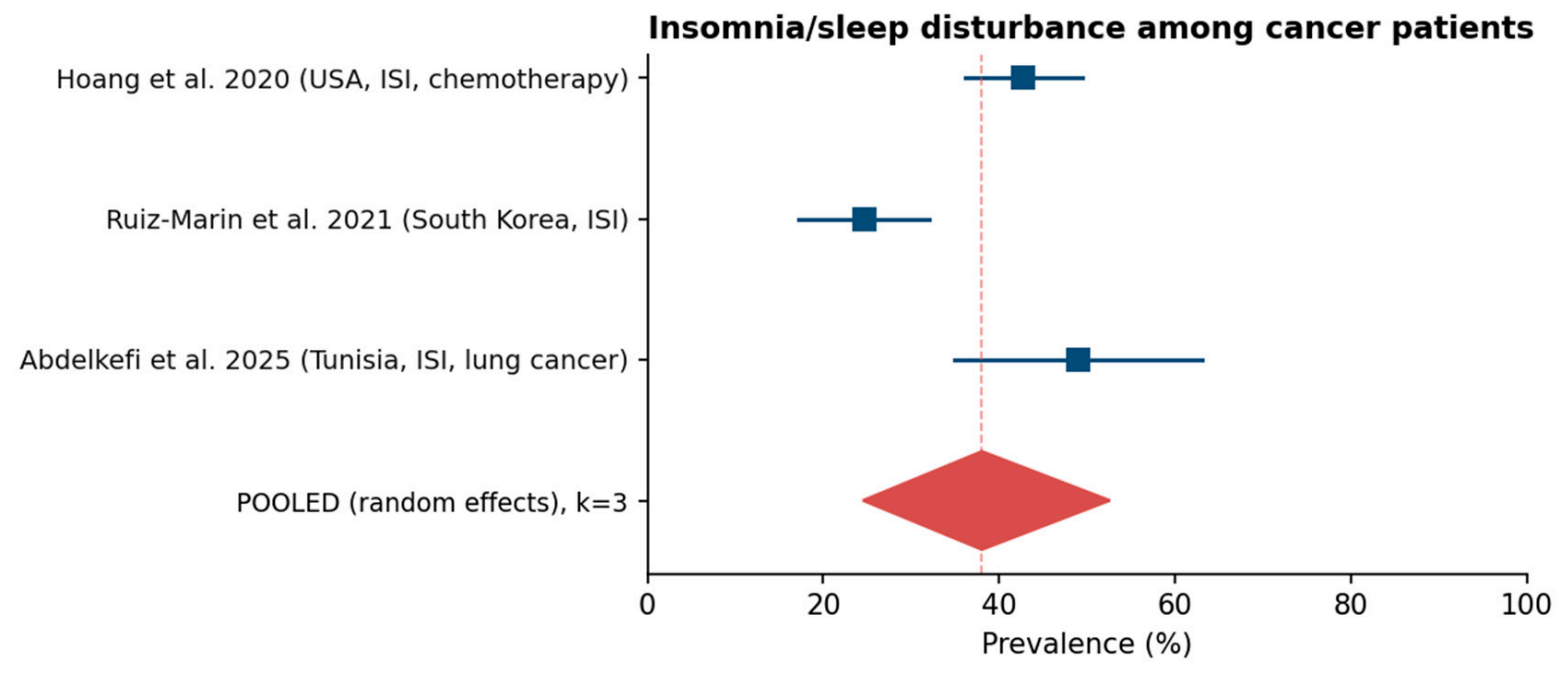
Illustrative forest plot of insomnia prevalence in oncology patients (random effects model; preliminary sample, k = 3) [30,31,32].

Three studies (total N = 392) reported insomnia prevalence using the Insomnia Severity Index in a United States chemotherapy cohort [30], a South Korean mixed cancer cohort [31], and a Tunisian lung cancer palliative cohort [32]. The pooled prevalence was 38.0% (95% CI: 24.5–52.6%; Q = 15.03, df = 2, *p* < 0.001; I^2^ = 86.7%; τ^2^ = 0.014) (Figure 4). Despite the shared instrument, thresholds differed slightly across studies (any insomnia versus moderate-to-severe insomnia specifically), a distinction that likely explains part of the observed spread and that a fully systematic review would need to standardise before pooling.

### 3.6. Suicidal Ideation

A single eligible study was identified reporting suicidal ideation prevalence using a defined instrument: a multicentre Chinese study of 509 women with advanced breast cancer, which found a single-item self-report suicidal ideation prevalence of 22.8% (116/509; 95% CI: 19.3–26.7%, Wilson score interval), with depression and emotional distress emerging as the strongest independent correlates in multivariable analysis [33]. As with the corresponding single-study outcome in our companion review of emergency medical services personnel, no pooled estimate is presented for an outcome represented by only one study; this figure is reported descriptively to establish the outcome as a priority for the planned systematic review, where it is expected that considerably more primary data—including studies of tumour types beyond breast cancer—will be available. This single-study finding must not be generalised beyond the specific sample from which it derives (women with advanced breast cancer in a Chinese multicentre setting); it says nothing, on its own, about suicidal ideation prevalence in oncology patients with other tumour types, at other treatment phases, or in other healthcare systems, and is presented here solely to flag an outcome that the planned systematic review must prioritise, not as evidence of a population-level rate.

## 4. Discussion

### 4.1. Principal Findings

Read alongside one another, the five outcome-level syntheses above tell a fairly consistent story: on self-report screening instruments, roughly two-fifths to just under half of patients in active cancer treatment or recent survivorship report clinically significant depressive or anxious symptoms, while PTSD and insomnia—assessed with somewhat different, and in the case of PTSD notably more conservative, instruments—cluster lower, at roughly one-sixth and two-fifths of patients, respectively. Every single pooled estimate, however, carried an I^2^ above 85%, meaning that in each case the great majority of observed variance across studies reflects genuine between-study differences rather than sampling error. This is the central empirical finding of the present work: not any one prevalence figure in isolation, but the near-uniform presence of very high heterogeneity across five conceptually distinct outcomes, assembled from an admittedly small and non-exhaustive sample of studies.

### 4.2. Relationship to Existing Meta-Analytic Evidence

The illustrative depression and anxiety estimates reported here (45.6% and 44.1%, respectively) sit well above the figures from the two most frequently cited interview-based syntheses—16.5% and 9.8% in Mitchell et al.’s 94-study meta-analysis [6], and 14% for depression in Krebber et al.’s subsequent synthesis of diagnostic interviews and self-report instruments combined [8]—but are broadly consistent with the upper end of the range Krebber et al. themselves reported when self-report cut-offs were used in isolation (up to 24% at a lenient HADS threshold) [8], and with the considerably higher figures reported specifically for lower- and lower-middle-income settings (21% and 18% for depression and anxiety, respectively) [7]. Every study contributing to our depression and anxiety estimates used a self-report screening instrument rather than a structured diagnostic interview, and the majority were conducted in South or Southeast Asian or lower-middle-income settings; both factors are independently associated with higher reported prevalence in the wider literature [7,8], and their combination in the present sample plausibly accounts for much of the gap between our figures and the classic interview-based benchmarks. A population-based Australian cohort assessed six months after diagnosis, by contrast, reported anxiety and depression prevalence considerably closer to our interview-based comparators [35], and a meta-analysis comparing long-term survivors with matched spouses and healthy controls found the excess risk relative to controls to be modest and, for depression specifically, non-significant [36]—a reminder that self-report screening prevalence at a single time point, of the kind assembled in the present sample, is not necessarily equivalent to a stable elevation in diagnosable disorder over the survivorship trajectory. Screening instrument scores are also known to run systematically higher than clinician-rated caseness even within the general population when compared against normative data [37], a further reason to expect self-report-based syntheses such as this one to sit above interview-based benchmarks such as Mitchell et al.’s [6]. Tumour type itself has separately been shown to move prevalence considerably, with lung and brain cancer patients consistently among the most affected and breast cancer patients among the least in large comparative samples [38], a pattern corroborated in an independent cross-sectional analysis of routinely collected UK oncology clinic data spanning multiple tumour sites [39]—a distinction the present sample, dominated by breast and mixed cancer cohorts, was not positioned to examine. Instrument-driven inflation of this kind is not unique to oncology: a broader synthesis of depression prevalence among general medical outpatients found comparably wide, instrument-dependent variation even outside the cancer setting [34], suggesting that some part of the heterogeneity documented here reflects screening instrument behaviour rather than any property specific to cancer populations. Our pooled PTSD estimate (17.0%) falls toward the upper end of the 5–15% range reported across instruments in the dedicated cancer PTSD meta-analysis by Swartzman and colleagues [11], again likely reflecting the inclusion of a paediatric, recently diagnosed subgroup with a markedly elevated point estimate. Our pooled insomnia figure (38.0%) is notably lower than the 60.7% reported in the large sleep disturbance meta-analysis by Al Maqbali et al. [10]; because that review’s substantially larger evidence base spanned a wider range of cancer types, treatment settings, and sleep disturbance definitions than our three-study sample, this discrepancy is better read as a limitation of the present preliminary synthesis than as a substantive disagreement about the underlying phenomenon.

### 4.3. Candidate Sources of Heterogeneity

Three explanations for the very high I^2^ values observed here merit explicit discussion, since each generates a testable prediction for the planned systematic review. First, instrument and threshold: depression and anxiety prevalence in the present sample varied by a factor of nearly two depending on whether a lenient HADS “borderline-or-above” cut-off or a stricter two-item screen was applied [23,25], a pattern entirely consistent with prior work showing prevalence estimates shifting by as much as threefold across instruments within the same underlying literature [8]. Second, treatment phase and time since diagnosis: the paediatric PTSD estimate in our sample was assessed within three months of diagnosis and was roughly three times the adult estimates drawn from longer-term survivor cohorts [27,28,29], mirroring the well-documented decline in psychological symptom prevalence over the treatment and survivorship trajectory reported elsewhere for both PTSD [11] and depression [9]. Third, country income setting: the two highest depression and anxiety estimates in our sample both derived from South Asian cohorts [23,25], consistent with the substantially elevated prevalence previously reported for low- and lower-middle-income countries relative to high-income settings [7]. A fourth, not examined in the present sample but well documented elsewhere, is patient demographic composition: race and ethnicity have been shown to independently predict post-traumatic stress symptom levels in breast cancer cohorts even after adjustment for clinical and socioeconomic covariates [40], and a systematic review restricted to breast cancer patients specifically found post-traumatic growth and post-traumatic stress to coexist far more often than a simple prevalence figure would suggest [41]. For insomnia specifically, longitudinal work following patients from diagnosis through 18 months indicates that new-onset sleep disturbance often persists well beyond the acute treatment period rather than resolving spontaneously [42], while a large North American oncology practice sample found sleep disruption prevalence and severity to vary systematically with treatment modality and disease stage [43]—both plausible contributors to the spread observed in our own three-study insomnia synthesis. None of these candidate explanations, our own four included, can be formally disentangled from the twelve-study sample assembled here; each requires the larger, moderator-coded dataset that only a fully systematic search can supply.

### 4.4. Implications for Clinical Screening

Whatever the precise pooled figure ultimately proves to be, every synthesis to date—the present preliminary one included—situates the burden of depression and anxiety among cancer patients well above general population rates, a conclusion robust enough to support continued investment in routine distress screening throughout the treatment pathway, consistent with existing psycho-oncology clinical guidance. The pattern observed here for PTSD and, more tentatively, for suicidal ideation carries a more specific implication: because both appear elevated in the early post-diagnosis period and around the time of receiving news of advanced or recurrent disease, screening protocols timed only at treatment initiation or at fixed survivorship intervals may miss the periods of peak risk for these particular outcomes. A screening cadence explicitly anchored to disease status transitions—new diagnosis, disease progression, treatment completion—rather than to calendar time alone may therefore warrant further evaluation. This is broadly consistent with existing clinical guidance recommending distress be treated as a “sixth vital sign” assessed at every clinical encounter rather than at fixed intervals alone [44,45], and with narrative reviews of breast cancer-specific depression epidemiology that have long argued for proactive rather than reactive screening models [46]. Meta-analytic evidence for psychosocial and psycho-oncologic interventions—both general [47] and cancer-specific [48]—further suggests that, once identified, a meaningful proportion of this symptom burden is amenable to treatment, strengthening the case for screening infrastructure capable of actually triaging patients into such interventions rather than merely documenting distress.

### 4.5. A Polish and Regional Perspective

None of the twelve studies identified in this preliminary exercise were conducted in Poland or in a directly comparable Central European healthcare setting—a gap that is itself informative, since it mirrors the broader underrepresentation of this region in English-language psycho-oncology reviews noted in our companion narrative synthesis on emergency medical services personnel. This gap is particularly salient given the authors’ own ongoing research programme in Polish psycho-oncology, which has examined vitamin D status and molecular tumour characteristics in Polish breast cancer patients and, in parallel work, the recognition of psychological distress by oncology nursing staff; the present synthesis is intended to provide international context for that programme [49] rather than to substitute for dedicated Polish prevalence data, which the planned systematic review will specifically seek to identify. Given the consistently documented association between depression, anxiety, and adverse oncological outcomes—including, in some analyses, cancer-specific mortality [50]—the absence of population-specific Polish prevalence estimates for depression and anxiety in oncology patients represents a genuine evidence gap with plausible relevance to the design of psychosocial oncology services nationally, and one the authors intends to address directly in forthcoming primary research.

### 4.6. Limitations

The limitations of this exploratory synthesis are substantial, and several bear directly on how its findings should—and should not—be used. Most fundamentally, this is a narrative review with an illustrative quantitative synthesis, not a systematic review, and the two should not be conflated: the twelve included studies were identified through a single-reviewer, non-exhaustive search rather than a systematic multi-database query following a predefined, reproducible search string across PubMed/MEDLINE, Scopus, Embase, PsycINFO, Web of Science, and CINAHL, so the assembled sample is a convenience sample and cannot be assumed representative of the full available literature; the sizeable gap between our depression and anxiety figures and the classic interview-based meta-analyses [6,8] is at least partly attributable to this non-representativeness rather than to a genuine shift in the underlying evidence. Given the breadth of the research question addressed here—prevalence of five distinct mental health outcomes across the full oncology population—a complete, systematic literature review stratified by disease phase, tumour type, and patient sex is the methodologically appropriate design for answering it definitively, and is precisely what the companion protocol referenced throughout this article commits the author group to undertaking next; the present work should be read as a scoping exercise that motivates and informs that undertaking, not as a substitute for it. The number of studies contributing to each outcome was small throughout—three for depression, four for anxiety, three for PTSD, three for insomnia, and one for suicidal ideation—and none reached the ten-study threshold conventionally required for meaningful publication bias testing or meta-regression [17,18], so the candidate explanations discussed in Section 4.3 remain plausible hypotheses rather than tested findings; readers should not treat them as established moderators. No formal, dual-rater risk-of-bias instrument—such as the Joanna Briggs Institute checklist for prevalence studies [13]—was applied to the included studies, and the pooled figures reported here are consequently unadjusted for study-level methodological quality; this is a substantive limitation, not a minor procedural gap, since undetected quality differences between studies could materially inflate or deflate any of the pooled estimates in Section 3. The back-transformation from the arcsine scale used a simplified approximation rather than the variance-weighted method recommended for definitive reporting [21]. Finally, several outcomes—most starkly suicidal ideation, represented by a single study of 509 women with advanced breast cancer—are supported by too little data even to characterise heterogeneity, let alone to estimate a stable pooled figure, and should be interpreted only as identifying a research priority, never as answering a clinical question about suicidal ideation prevalence in oncology patients generally.

## 5. Conclusions

This narrative review with preliminary quantitative synthesis found that, across a small but internationally varied sample of twelve studies, illustrative pooled prevalence estimates for depression (45.6%) and anxiety (44.1%) among cancer patients considerably exceeded the benchmarks established by classic interview-based meta-analyses, while PTSD (17.0%) and insomnia (38.0%) occupied an intermediate position relative to prior syntheses—all four estimates carrying very high heterogeneity (I^2^ > 85%). Rather than treating any of these figures as a settled answer, the more useful contribution of this preliminary work is arguably the pattern itself: the consistent finding, replicated across five conceptually distinct outcomes, that instrument choice, treatment phase, and country income setting each plausibly move prevalence estimates by a substantial margin, and that no single pooled figure drawn from a handful of studies should be presented to clinicians or policymakers as definitive. Given the breadth of the underlying question and the clinical stakes involved, the authors agree that a complete, systematic review—stratified by cancer type, treatment phase, and patient sex, and incorporating formal risk-of-bias assessment—rather than further narrative work of the present kind, is the necessary next step; this exploratory synthesis is offered as motivation and groundwork for that undertaking, not as a substitute for it. The fully systematic review the authors have planned and protocolled will directly test these candidate explanations through moderator-coded meta-regression once a database-searched, dual-reviewer-screened evidence base of sufficient size is assembled—with particular attention, given the gap identified in Section 4.5, to Polish and Central European primary studies that remain essentially absent from the current international synthesis literature on psychological morbidity in oncology populations.

## Data Availability

The data extraction spreadsheet and analysis code supporting this preliminary synthesis are available from the corresponding author upon reasonable request.
